# Antimicrobial Potential of Brassica oleracea Extracts (White and Broccoli) and Their Resistance Compared to Doxycycline Against Gram‐Positive and Gram‐Negative Bacteria

**DOI:** 10.1002/fsn3.4345

**Published:** 2024-07-28

**Authors:** Meisam Rahmani, Akbar Esmaeili, Mahboubeh Taherkhani

**Affiliations:** ^1^ Department of Basic Science, Central Tehran Branch Islamic Azad University Tehran Iran; ^2^ Department of Chemical Engineering, North Tehran Branch Islamic Azad University Tehran Iran; ^3^ Department of Chemistry, Takestan Branch Islamic Azad University Takestan Iran

**Keywords:** *B. oleracea* extract (white and broccoli), doxycycline, resistance, strain

## Abstract

*Brassica oleracea* L is low in carbohydrates and fiber, making it ideal for low‐carb diets. But they are rich in vitamins, minerals, and other essential nutrients. The present article investigated the antimicrobial effect of *B. oleracea* extract (white and broccoli) (BOE‐WB) on Gram‐positive and Gram‐negative strains and its resistance to doxycycline. BOE‐WB was used as the soaking method. It was analyzed by gas chromatography–mass spectroscopy (GC–MS), high‐performance liquid chromatography (HPLC), and 2,2‐Diphenyl‐1‐picrylhydrazyl (DPPH) methods. The samples were prepared with three concentrations. It used Mueller Hinton Agar culture medium of fresh bacteria. It was incubated for 24 h in a 37°C incubator. It identified 33 compounds. For BOE‐W, 77% of the compounds are oxygenated, while for BOE‐B, this percentage is 79%. For BOE‐B, 79% had oxygenated compounds. BOE‐WB has significant antibacterial effects on *Pseudomonas aerogenosa* and *Bacillus cereus*. The lethal impact of BOE‐WB on strains is very close to that of doxycycline, and it can be introduced as a new antimicrobial drug to the medical world. The research shows that the percentage of oxygenated compounds in foods containing BOE‐B is much higher than that of BOE‐W. It has a significant impact on the antioxidant effect. Foods containing BOE‐W have a high percentage of azo compounds and sulfur. One of the benefits of sulfur in the body is disinfecting the blood. In addition, sulfur increases the body's resistance to bacteria.

## Graphical abstract

Resistance effect of the strains on the doxycycline for the bacterial resistance of the body. 91.20% of BOE‐B had oxygenated compounds. BOE‐B had strong antioxidant properties. Gram‐negative bacteria were on *P. aerogenosa*, *S. enterica*, and *E. coli*.

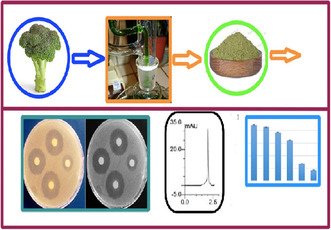

## 1 INTRODUCTION

Low‐carb diets are very different. Most have <150 g of carbohydrates daily, and some reach 20 g daily. Eating more vegetables is always a great idea, whether on a low‐carb diet or not. The Brassicaceae family consists of 350 genera and about 3500 species. It includes a wide range of garden products (Cartea et al., 2010). Studies show that BOE may reduce insulin resistance in type 2 diabetes. It is also thought to protect against several types of cancer, including prostate cancer. *B. oleracea* extract (white and broccoli) contains vitamins, minerals, and phytochemicals and is a superfood. Unlike other cruciferous vegetables, red cabbage is unique because it includes a large amount of anthocyanins. Anthocyanin is not only considered an antioxidant nutrient but also an anti‐inflammatory nutrient. BOE‐B is rich in vitamin K, C, and B complex. Red cabbage is also an excellent source of minerals such as manganese, iron, magnesium, and potassium. The main nutritional components of Brassicaceae vegetables are carbohydrates, proteins, vitamins, and minerals (Manchali et al., 2012). It becomes an essential component of a low‐fat and heart‐friendly diet. In addition to macro and micronutrients, cruciferous vegetables are rich in bioactive and non‐nutritive phytochemicals. It is associated with a reduced risk of several chronic diseases (Liu, 2004). Cruciferous vegetables have received considerable attention in recent years due to their contribution to improving health and preventing cancer and cardiovascular diseases. Extensive epidemiological studies have shown an inverse relationship between the consumption of cruciferous vegetables and the risk of various types of cancer. Cruciferous vegetables have become increasingly important in cancer (Manchali et al., 2012). Reducing platelet aggregation, lowering blood pressure, modulating the synthesis and absorption of cholesterol and anti‐inflammation play a role in preventing cardiovascular diseases (Liu, 2013).

Liu reported on evaluating the antibacterial activity of BOE against *Salmonella typhimurium* (Liu, 2013). In 2023, they surveyed to identify possible medicinal sources of BOE‐WB (Jang et al., 2015). Muntean et al., from Romania, reported the medicinal properties of four Romanian plants, such as BOE‐WB and black tarp (Muntean et al., 2021). Kyung et al. described hydrolysis products on 15 Gram‐positive and Gram‐negative bacteria (Kyung & Fleming, 1997). Dias et al. performed on several vegetable products with the highest antimicrobial activity. It showed *S. aureus* resistant to methicillin isolated from patients with diabetic ulcers (Dias et al., 2014). Jadoun et al. reported antimicrobial effects on radish seeds with the aim of antimicrobial solid activity on several strains (Jadoun et al., 2016).

**SCHEME 1:**
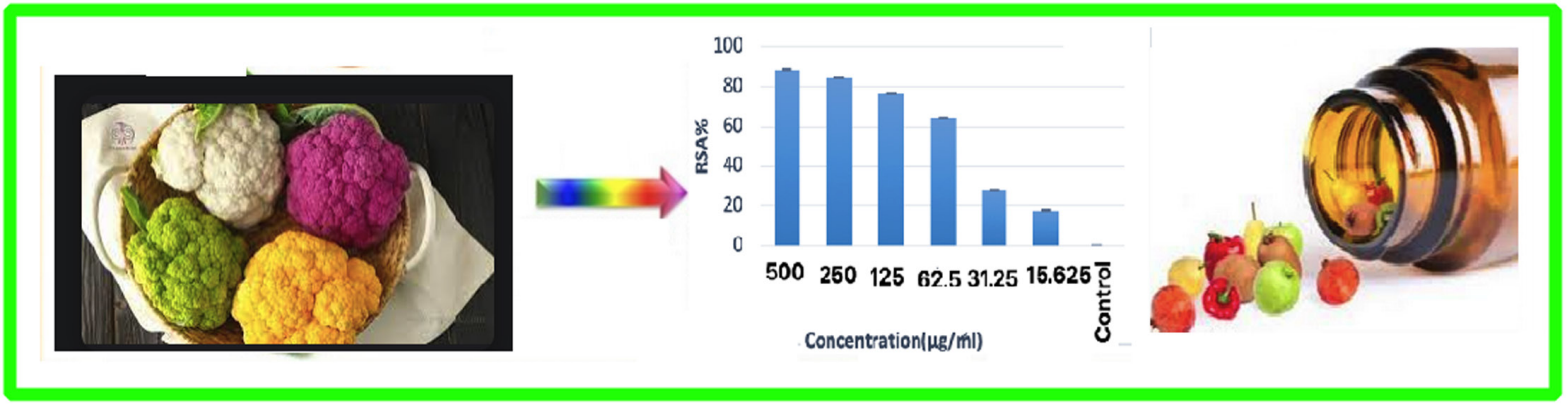
Image of processes for BOE system.

The present research investigates the effects of BOE‐WB (Scheme 1). It is based on three Gram‐positive and three Gram‐negative strains. Finally, it compares its resistance with doxycycline and proposes an extract as a suitable alternative to this drug. Three Gram‐positive [*Streptococcus mutans* (ATCC 35668), *Staphylococcus aureus* (ATCC 29213), and *Bacillus cereus* (ATCC 14579)] and three Gram‐negative [*Escherichia coli* (ATCC® 25922™), *Pseudomonas aeruginosa* (ATCC 27853), and *Salmonella enterica* (ATCC 8270)] used in the research.

## 2 EXPERIMENTAL

### 2.1 Collecting of *B. oleracea* extract (white and broccoli) (BOE‐WB)

The researched plant was harvested in December 2022 from the 5‐km fields of Qazvin city located in the northwest of Canada, wholly crushed, and ground and extraction were performed on it.

### 2.2 BOE‐WB extraction

BOE‐WB was purchased from a herbal shop. Hundred grams of BOE‐WB was ground, placed in a beaker, soaked for 24 h, then filtered and distilled under vacuum (yield: 2.5% (w/w)). For further analysis, it was kept in a dark glass container in the refrigerator (4°C).

### 2.3 DPPH method

Evaluation of the antioxidant properties of essential oils was also done using the DPPH method. In this method, the ability of essential oils to give electrons to DPPH was evaluated by changing the color of the DPPH methanol solution (from purple to yellow). Also, BHT, BHA, and TROLOX were used as standard control, and the results obtained from essential oils were compared with these substances as positive control.

The tests related to essential oils and standard samples were repeated three times to minimize test errors and ensure the results. After averaging the data, the results of each test were reported. The main advantage of DPPH reagent is that it has sufficient sensitivity and can measure a large number of samples in a short time. The extract was mixed with methanol, and different essential oil dilutions were prepared.

Ten milligrams of DPPH was made up to volume in a 250‐mL flask with methanol. Fifty microliters of different dilutions of the essential oil sample was mixed with 5 mL of DPPH solution. The resulting mixtures were kept in the dark for 30 min at the temperature of the laboratory. We used Trolox (1 mM), BHT (1 mM), and BHA (1 mM) as stable antioxidants. Specifically, BHA at 1 mM concentration corresponded to 1.8 mg/10 mL, BHT at 1 mM concentration corresponded to 2.2 mg/10 mL, and Trolox at 1 mM concentration corresponded to 2.5 mg/10 mL.

Absorption at 517 nm was measured and recorded using a spectrophotometer, and IC_50_ was obtained by plotting the antioxidant changes curve against the concentration. The radicalization activity of the essential oil is calculated by the following formula and based on the percentage of DPPH inhibition:

$$\text{Inhibition percentage}\;\left(\mathrm{IP}\right)=\left[\left(\mathrm{AB}–\mathrm{AA}\right)/\mathrm{AB}\right]\times 100$$

AB, the absorbance value of blanks, was checked after 30 min, and AA, the absorbance value of the sample, was checked after 30 min. The value of IC_50_ indicates a concentration of the compound that causes 50% inhibition in the radical capacity. The value of IC_50_ was determined by linear correlation analysis obtained from the RSC (IP) values in sample concentrations, and the results obtained were compared with the positive control (Esmaeili et al., 2009; Masoumi & Esmaeili, 2020b; Naderi & Esmaeili, 2020, 2021; Rajabi & Esmaeili, 2020; Salarieh et al., 2023).

### 2.4 Antibacterial activity

Samples (extract) were prepared with 1000, 500, and 250 μg mL^−1^ concentrations. Sterile UV rays were used, and fresh bacteria were cultured in a Mueller Hinton agar culture medium (with OD 0.1 turbidity in pure physiological serum). It was mass cultured with a clean swap. A volume of 30 μL of each concentration was loaded onto a blank disk. Along with an ampicillin antibiotic disk (with an attention of 25 μg per disk) as a positive control, they were placed in the agar medium with suitable intervals. The samples were then placed in an incubator at 37°C for 24 h, and the diameter of the growth inhibition zone was measured using a ruler.

### 2.5 Microbial methods

In this experiment, the antimicrobial effect of the extracts was performed on the following bacteria. *E. coli* (ATCC® 25922™), *P. aeruginosa* (ATCC 27853), *S. enterica* (ATCC 9270), *S. aureus* (ATCC 29213), *S. mutans* (ATCC 35668), and *B. cereus* (ATCC 14579). For microbiology methods, standard methods were used. The recorded result of each is the average of three tests. A diffusion test was used to study the antimicrobial effects, and the plate method disk was used among the diffusion methods (Esmaeili & Mohabi, 2014; Liu et al., 2023).

Samples (extracts) were prepared with 1000, 500, and 250 μg mL^−1^ concentrations. They were sterilized using UV–Vis in Mueller Hinton agar culture medium of freshly cultivated bacteria (with turbidity OD = 0.1 in physiological serum sterile) and mass cultured with clean swap. Then, 30 μL of each concentration was loaded on an empty disk and placed in the agar medium with an ampicillin antibiotic disk (with an attention of 25 μL per disk) as a positive control at suitable intervals and for 24 h. They were incubated in a 37‐degree incubator, and then, the diameter of the lack of growth was measured by a ruler.

### 2.6 GC–MS

To investigate terpenoid compounds and BOE, GC–MS was used to separate and determine the percentage of each essential oil component. Extract evaluation was done with an Agilent 5977A device equipped with a quadrupole MS detector (MD800) and three biological replicates. A silica column (Chrompak) with a length of 50 m, a diameter of 0.25 mm, and a particle size of 0.25 micrometers was used for the present study. The mentioned column was a capillary column made of phenyl (5%) and dimethylpolysiloxane (95%), and the injector temperature (Shahrokhi & Esmaeili, 2023; Tahmasebi & Esmaeili, 2023) was 280 degrees Celsius. The temperature of the column reached 120 degrees Celsius at a rate of 4°C per minute, then 200°C at a rate of 2°C per minute, and finally 280°C at a rate of 25°C per minute.

The MS ionization energy was set to 70 electron volts, and the ionization temperature was set to 280°C. The carrier gas was helium and flowed at 1 mL per minute. One microliter of the sample is injected for each measurement. The samples were first diluted 1:100 with n‐hexane for essential oil and then injected into the device. Peaks with the software Mass lab were analyzed at one scan per second. Identification of extract compounds by calculating the Quartz coefficient (which was done by injecting normal n‐alkane hydrocarbons (Davies, 1990) (c11–c28) under the same conditions as the injection of essential oils) and checking the mass spectrum of the compounds and comparing them with the mass spectra suggested by GC–MS device libraries and also by comparing mass spectra of standard compounds available in Adams library (Ghannadi & Amree, 2002) were compared. The correctness of the compounds was confirmed by calculating the Quartz coefficient and using n‐alkane (c11–c28) based on the following formula (Khodaei & Esmaeili, 2019, 2020; Mahmoudi et al., 2022, 2023; Masoumi & Esmaeili, 2020a, 2020b).

### 2.7 HPLC

The mobile phase consisted of acetonitrile and water in the ratio (18:88 v/v) with a flow rate of 0.5 mL min^−1^, and the analysis was performed by reverse phase and isocratic chromatography. The peaks were detected at a wavelength of 254 nm, and a volume of 20 μm was injected into the HPLC device (Yang et al., 2020).

### 2.8 Statistical analysis

The experiments were repeated three times, and their results were reported as mean ± SD. The statistical analysis of the data was done with a one‐way analysis of variance using SPSS software, and Tukey's test was used to compare the mean of the groups. *p* < .05 was considered statistically significant.

## 3 RESULT AND DISCUSSION

### 3.1 *B. oleracea* extract (white and broccoli) (BOE‐WB)

BOE is one of the most popular vegetables and a member of the cabbage family. It has many properties for the body and is readily available throughout the year. These vegetables were first cultivated in Europe, and today, China and India have the most significant production in the world. BOE is a plant species in the Brassicaceae family that includes many common cultivars. It protects body cells against the harmful effects of free radicals and inflammatory attacks. Like other cruciferous vegetables, the food contains abundant glucosinolates and isothiocyanates. It slows down the growth of cancer cells. Laboratory studies have shown that glucosinolate and isothiocyanate can protect the body against colon, lung, breast, and prostate cancer (Muntean et al., 2021). BOE contains 4 g of digestible carbohydrates per serving. Rich in vitamins C and K, it may reduce insulin resistance and help prevent cancer.

Several studies showed that cruciferous vegetables have good biological activity based on the synergistic effect of glucosinolates, polyphenols, and triterpenes. It can include anticancer (Hormozi & Esmaeili, 2019), antioxidant (Esmaeili et al., 2015), anti‐inflammatory (Morales‐López et al., 2017), and cardioprotective vegetables (Favela‐González et al., 2020). Glycosylation of triterpenes is one of the plant's defense mechanisms. It leads to the synthesis of triterpene. It has good antimicrobial (antibacterial, antiviral, antifungal) (Hu et al., 2004), antiparasitic, anti‐inflammatory, and other anticancer properties, such as anti‐obesity (Favela‐González et al., 2020), liver protection (Morales‐López et al., 2017), and digestive security (Manchali et al., 2012) and immunomodulatory (Hu et al., 2004), which was observed by testing some Brassicaceae extracts.

Aqueous extract of cauliflower can help changes in blood vessels. Specifically, oxidative stress‐induced endothelial dysfunction reduces the bioavailability of nitric oxide. Endothelial dysfunction will affect the initial stage of cardiovascular disease. This research uses it to treat blood vessels, resulting from the plant extract's antioxidant properties. The results of the GC–MS show the three compounds: decanoates and carboxylate. It reported the highest percentage.

The antibacterial test results on four strains gave the following results: *E. coli*, with a halo diameter of 18 mm in the concentration of 500 μg mL^−1^ and 16 mm in the concentration of 250 μg mL^−1^. *S. mutans*: with a halo diameter of 17 mm in the concentration of 500 μg mL^−1^ and 15 mm in the concentration of 250 μg mL^−1^. *S. epidermidis*: with the diameter of the halo, the number of non‐growths at the concentration of 500 μg mL^−1^ was 16 mm, and at the concentration of 250 μg mL^−1^, it was 14 mm. *P. aeruginosa*: With the diameter of the halo, he reported a lack of growth in the concentration of 500 μg mL^−1^, 20 mm, and the concentration of 250 μg mL^−1^, 17 mm. Table 1 shows the antibacterial test results of BOE‐WB for Romanian and recent research.

**TABLE 1:** Antibacterial test results of BOE‐WB for Romanian and recent research.

Microorganism	Romanian family		Recent research	
	BOE‐W	BOE‐B	BOE‐W	BOE‐B
*S. mutans*	15.00 ± 0.00	15.33 ± 0.58	9.00	12.00
*S. aureus*	14.67 ± 0.58	15.00 ± 0.00	0.00	0.00
*S. pyogenes*	15.33 ± 0.58	15.67 ± 0.58	–	–
*E. coli*	15.67 ± 0.58	16.00 ± 0.00	9.00	10.00
*P. aeruginosa*	15.00 ± 0.00	15.00 ± 0.00	15.00	14.00
*C. albicans*	7.33 ± 0.58	7.33 ± 0.58	–	–

In another research, three BOE species were conducted to identify the medicinal properties of four Romanian plants. Cruciferous vegetables are a rich source of dietary antioxidants. Climatic conditions, agricultural practices, storage conditions, etc., influence these vegetables' antioxidant content. (Muntean et al., 2021). UV‐vis technique was used to evaluate the antioxidant effect. The analyzed extracts have good antioxidant activity (0.97–1.13 mmol). Table 2 shows antimicrobial activity using the disk diffusion method. Most of the strains showed symmetrical and moderate sensitivity to BOE‐WB.

**TABLE 2:** Antibacterial activity of BOE‐WB.

Concentration (μg mL^−1^)	*E. coli* (ATCC 25922)			*S. aureus* (ATCC 29213)			*P. aeruginosa* (ATCC 27853)			*S. mutans* (ATCC 35668)			*S. enterica* (ATCC 9270)			*B. cereus* (ATCC 14579)		
	A	B	C	A	B	C	A	B	C	A	B	C	A	B	C	A	B	C
BOE‐W	9	7	0	0	0	0	15	12	0	9	7	0	12	10	8	11	10	9
BOE‐B	10	9	8	0	0	0	14	11	0	12	10	3	12	10	8	15	11	9
Doxycycline	30	28	25	36	32	30	35	32	27	16	18	17	26	26	24	26	24	23
Ampicillin		20			15			20			20			15			15	

*Note*: A: 1000, B: 500, C: 250.

### 3.2 DPPH assay

**FIGURE 1:**
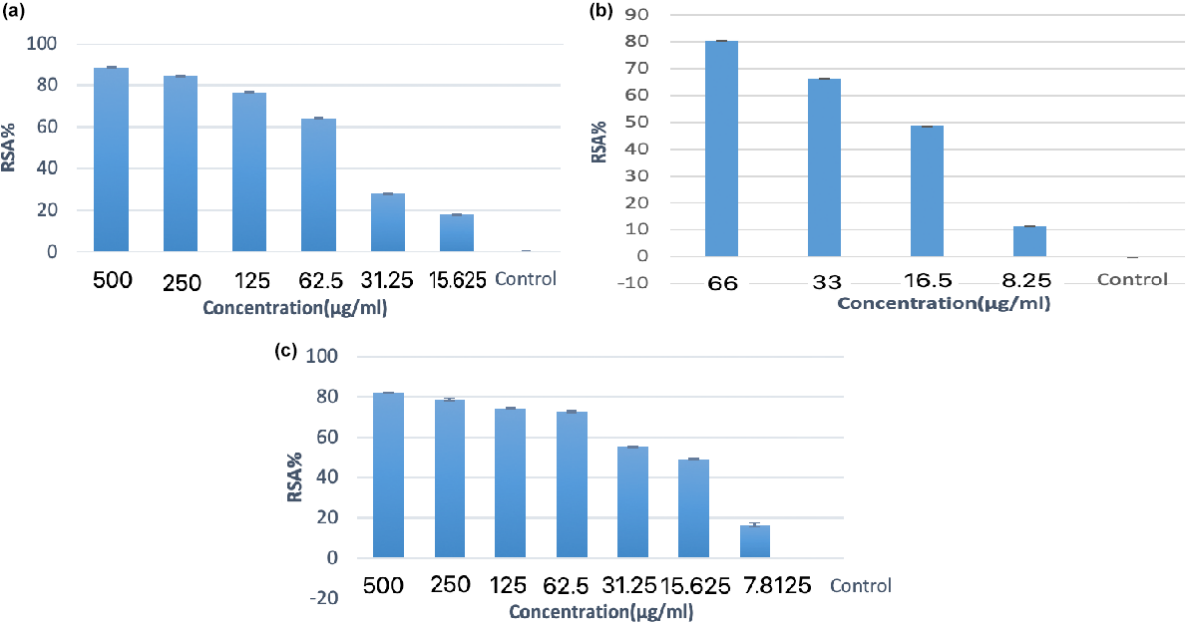
DPPH assay (a) BOE‐W, (b) Vitamin C, (c) BOE‐B.

In Asia, BOE was used for the first time many years ago, around 600 BC, as a food ingredient. But it is also used in many Italian, Spanish, Turkish, and French dishes today. BOE first arrived in America in the mid‐16th century and was primarily used for garnishing food. According to recent research results, the consumption of crops such as BOE is related to the prevention of chronic diseases, including cardiovascular diseases, diabetes, neurological disorders, and various types of cancers. According to what is published in reliable scientific sources, A serving of approximately 100 g of raw BOE has a nutritional value of 25 calories and more than 5 g of carbohydrates. BOE‐W water extract in concentrations (500, 250, 125, 62.5, 31.25, 15.62, 7.812) μg mL^−1^ significantly had the highest percentage (88.56%) in the attention of 500 μg mL^−1^ (Figure 1a).

**FIGURE 2:**
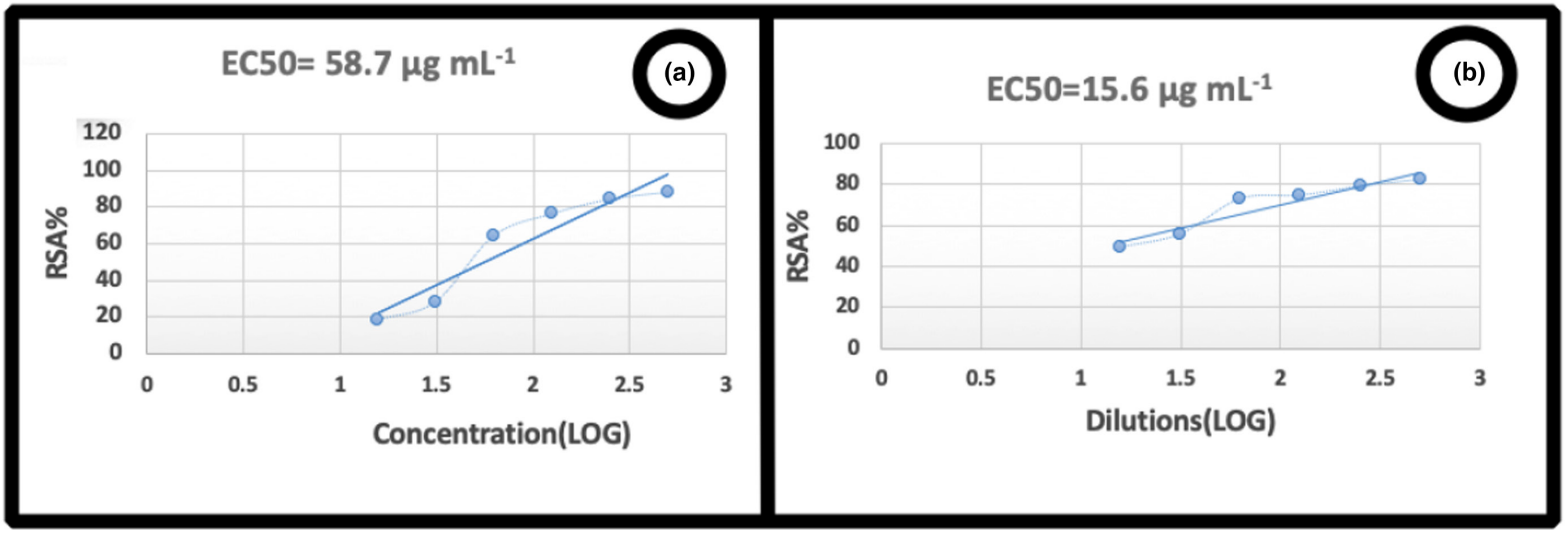
DPPH radical scavenging activity of (a) BOE‐W, (b) BOE‐B.

The highest percentage of Vitamin C was reported as the extract control at a concentration of 66 μg mL^−1^ compared to 80.54 μg mL^−1^. This test used Vitamin C as a control (Figure 1b). BOE water extract in concentrations (500, 250, 125, 62.5, 31.25, 15.62, and 7.812) μg mL^−1^ significantly had the highest percentage (82.35%) at a concentration of 500 μg mL^−1^ (Figure 1c). DPPH radical removal activity, BOE‐WB, is shown linearly in Figure 2a,b. These findings indicated that the extract's DPPH free radical inhibition activity was dose‐dependent. In addition, a stable suspension is achieved by absorbing the quinoid chemical. It is produced when the phenol group in phenols is oxidized on the surface of nanoparticles. In general, phenolic chemicals have a direct role in antioxidant activity. For this reason, their redox properties enable them to act as reducing agents. Plant phenols have antioxidant properties (Al Talebi et al., 2023).

The research that discussed BOE (Al Talebi et al., 2023; Esmaeili & Gholami, 2015; Zhang et al., 2023) has the highest percentages of 75.2, 68.01, 53.52, and 36.04 in 200, 100, 50, and 25 μg mL^−1^ concentrations. While in the research conducted by us in the present study, BOE in concentrations of 500, 250, 125, 62.5, 31.25, 15.62, and 7.81 μg mL^−1^ has the highest percentages of 82.35, 78.94, 74.55, 72.82, 55.59, and 49.11. In conclusion, the antioxidant property of the present study broccoli is higher according to the climate.

### 3.3 Antibacterial activity of white cauliflower and broccoli and doxycycline

The investigations carried out in this research showed the antibacterial effects of the investigated plant extracts (Table 2). BOE‐WB extract has sound germicidal effects on Gram‐negative and Gram‐positive bacteria, except for *Staphylococcus aureus* (Gram‐positive) bacteria, which did not affect this strain.

Among Gram‐negative bacteria, white cabbage and BOE had the most excellent germicidal effect on *P. aerogenosa*, *S. enterica*, and *E. coli*, respectively. The result is that BOE‐W has a more significant germicidal impact on *P. aeruginosa* strain than BOE‐B. BOE‐W had an equal ratio (microbicide) in the *S. enterica* strain. BOE compared to white BOE‐B with more germicidal effect in *E. coli* strain. The antibiotics under study (Doxycycline) in the order of the most excellent germicidal effect were *P. aerogenosa*, *E. coli*, and *S. enterica*, respectively.

Among Gram‐positive bacteria, BOE‐WB extract had the most excellent germicidal effect on *B. cereus, S. mutans*, and *S. aureus*, respectively. Compared to BOE‐W, BOE‐B had a more germicidal effect on *B. cereus* (lowest bacterial resistance) and *S. mutans* (highest bacterial resistance) strains. BOE‐WB had no effect on *S. aureus* strain. The antibiotic under study, doxycycline, killed *S. aureus*, *B. cereus*, and *S. mutans* (Esmaeili et al., 2006).

**FIGURE 3:**
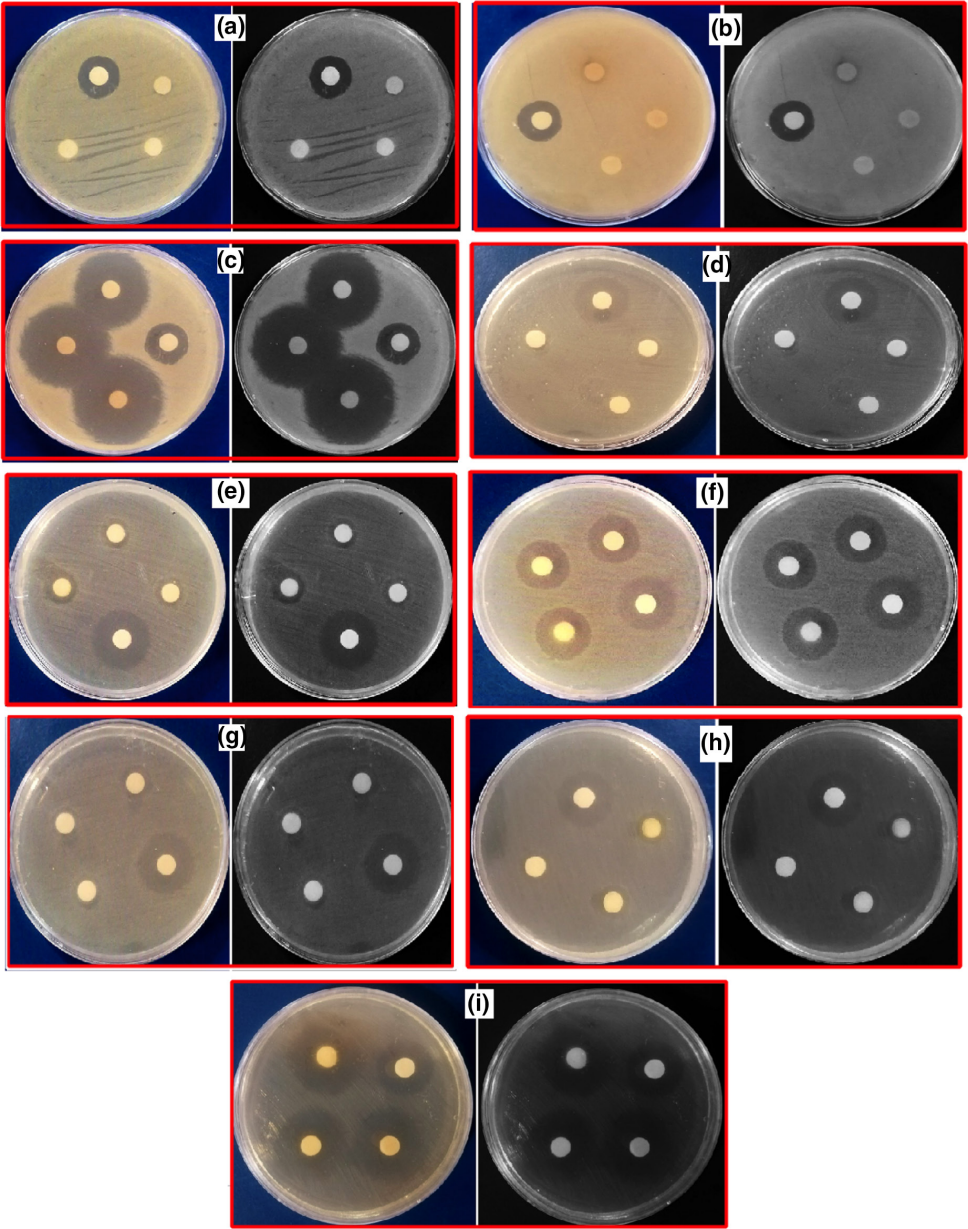
Mueller Hinton Agar culture medium for bacteria *S. aureus*: (a) 0.1 BOE‐W, (b) 0.2 BOE‐B, (c) 0.3 doxycycline, *S. mutans*: (d) 0.1 BOE‐W, (e) 0.2 BOE‐B, (f) 0.3 doxycycline, *B. cereus*: (g) 0.1 BOE‐W, (h) 0.2 BOE‐B, (i) 0.3 doxycycline.

Among Gram‐positive bacteria, BOE‐WB extract had the most excellent germicidal effect on *B. cereus, S. mutans*, and *S. aureus*, respectively. Figure 3 shows the Mueller Hinton Agar culture medium for bacteria *S. aureus*, *S. mutans*, and *B. cereus* with 0.1 BOE‐W, 0.2 BOE‐B, and 0.3 doxycycline, respectively. Compared to BOE‐W, BOE‐B had a more germicidal effect on *B. cereus* (lowest bacterial resistance) and *S. mutans* (highest bacterial resistance) strains. BOE‐WB had no effect on *S. aureus* strain. The antibiotic under study (Doxycycline) had the most significant effect of killing *S. aureus*, *B. cereus*, and *S. mutans*, respectively. In a research conducted on Romanian BOE‐WB (Muntean et al., 2021), the result compared to the present study BOE‐WB is as follows: Romanian BOE‐WD bactericidal in *S. mutans* (Gram‐positive) strain reported 15 and 15.33 mm, respectively. In the present study, BOE‐WB reported 9 and 12 mm. Romanian BOE‐WB reported 14.67 mm and 15 mm in (Gram‐positive) *S. aureus* strain, respectively. In contrast, the present study BOE‐WB did not affect the *S. aureus* strain. Romanian BOE‐WB in *E. coli* strain (Gram‐negative) reported 15.67 and 16 mm, respectively, while the present study BOE‐WB reported 9 and 10 mm.

Bactericidal Romanian BOE‐WB was reported in *P. aeruginosa* (Gram‐negative) strain equal to 15 mm. At the same time, present study, BOE‐WB reported 15 and 14 mm in *P. aeruginosa*. On doxycycline, the antibiotic was Gram‐negative strains *E. coli*, 30 mm, *P. aeruginosa*, 35 mm, and *S. enterica*, 26 mm. The Gram‐positive strains of *S. aureus*, 36 mm, *S. mutans*, 19 mm, and *B. cereus*, 26 mm.

According to the reports from past research and this research, BOE‐WB has good antioxidant properties and good antibacterial results on all types of Gram‐positive and Gram‐negative bacteria, considering the resistance of bacteria to antibiotics. According to the effects of Romanian and Iraqi, and the present study BOE‐WB, according to the climatic conditions of each country, each country can have a suitable herbal substitute with doxycycline.

### 3.4 GC–MS analysis

They are naturally present in small amounts in food plants—substance components such as long‐chain hydrocarbons, steroids, alkaloids, alcohols, amines, and GC‐nitro compounds (Esmaeili, 2012). Tables S1 and S2 (File S1) show the percentage composition family of the BOE‐W and BOE‐B compounds after injecting the extract prepared by hexane into the GC–MS machine, the chromatogram of the section. It is based on the Coates index, and the compounds in the White cauliflower and BOE were obtained by examining and studying the chromatogram and mass spectra obtained from GC–MS. Table 3 shows the highest percentage of BOE‐WB compounds. According to Table 4, 32.42% of BOE‐W and 91.20% of BOE‐B were found to have oxygenated compounds. BOE‐WB has antioxidant properties. 30% of the compounds were identified in BOE‐W. It was recognized as monoterpene, 32.87% (BOE‐W), and 6.41% (BOE‐B) as sulfide and Silone family, 31.66% (BOE‐W), and 2.37% (BOE‐B) as azo compound family, of the compounds were identified. Figures S1 and S2 (File S1) show the diagram of GC–MS analysis of BOE‐WB. The presence of many oxygenated groups in BOE‐B neutralizes the reduction of free radicals. According to the antioxidant reaction, BOE‐B is more than BOE‐W. Surface materials containing BOE‐B are used as food supplements. It has high antioxidants. As oxygen is essential for plant respiration, storing oxygenated citrus fruits causes anticancer. Sulfites and sulfur compounds are abundant in BOE‐W. It is also found in many foods and beverages. Food is used to maintain and enhance its abilities. Sulfates inhibit microbial growth and, therefore, prevent food from being eaten. It also helps certain foods retain their natural color for more extended periods. While these compounds are added to many foods, they occur naturally and may become concentrated during preparation. Due to its unique properties for health, nutrition, and pleasant taste, it has a special place in healthy foods. BOE contains essential nutrients such as vitamin C, vitamin K, fiber, folate, potassium, and beneficial antioxidants such as sulforaphane that can help improve health. In addition, some studies have shown that BOE consumption can enhance the prevention of heart disease, cancer, diabetes, and other chronic diseases (Amand & Esmaeili, 2020; Esmaeili & Haseli, 2017).

**TABLE 3:** The highest percentage of BOE‐W and BOE‐B compounds.

Compounds	(%)	
	BOE‐W	BOE‐B
(2‐Methyl‐but‐3‐enyl‐2‐oxy)‐dimethyl‐silane	12.61	
Palmitic acid	6.98	
Ethylhexyl isophthalate	6.14	
Nonacosane	5.01	18.95
Octadecatrienal		10.72
Nonacosanone		9.92
Linolenic acid		8.60
Hexadecanoic acid		5.48
Benzyl benzoate		5.41

**TABLE 4:** Time of keeping significant peaks.

Sample	Compound	Time
BOE‐W	Peak 1	2.001
	Peak 2	2.460
BOE‐B	Peak 1	1.960
	Peak 2	2.413
	Peak 3	3.348
	Peak 4	3.669
Doxycycline	Peak 1	2.404
	Peak 2	2.669
	Peak 3	2.967

### 3.5 HPLC analysis

Although BOE also contains protein, like most vegetables, it is technically high in carbohydrates.

In this method, it is possible to quantitatively and qualitatively identify the mixture of compounds by using the inhibition time and the area under the peak. In white cauliflower, compound X enters in 1.5 min. At the sharp rise of 2.001 min, it leaves at 2.40 min while maintaining the peak time. Y composition enters in 2.40 min. At the bank, it is approximately 2.46 min while maintaining the peak time. It leaves at 3.25 min. In broccoli, compound X enters in 1.5 min. At the sharp peak of 1.960 min, it lasts 40 min while maintaining the peak time. Y composition enters in 2.40 min. At the rise, it is approximately 2.41 min while maintaining the peak time, it leaves at 3.25 min. In doxycycline, compound X was introduced at 2.5 min. In a sharp peak of 2.699 min, keeping the peak time, it exits at 2.90 min, and combination Y enters at 2.96 min. It leaves at 4, saving the same peak time, according to the arrival and departure times for the samples (Abadi & Esmaeili, 2022; Mahmoudi et al., 2023; Shahrokhi & Esmaeili, 2023; Tahmasebi et al., 2022).

**FIGURE 4:**
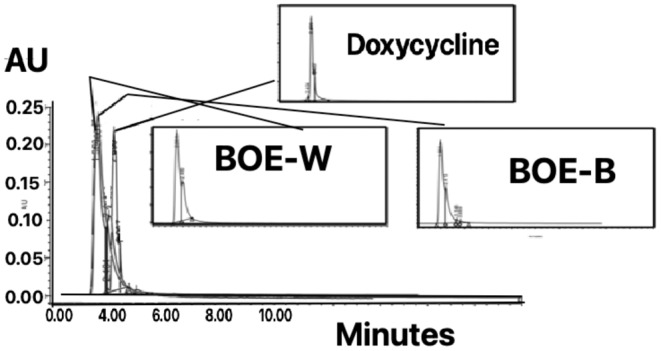
HPLC general spectrum for sample BOE‐W, BOE‐B, doxycycline.

**FIGURE 5:**
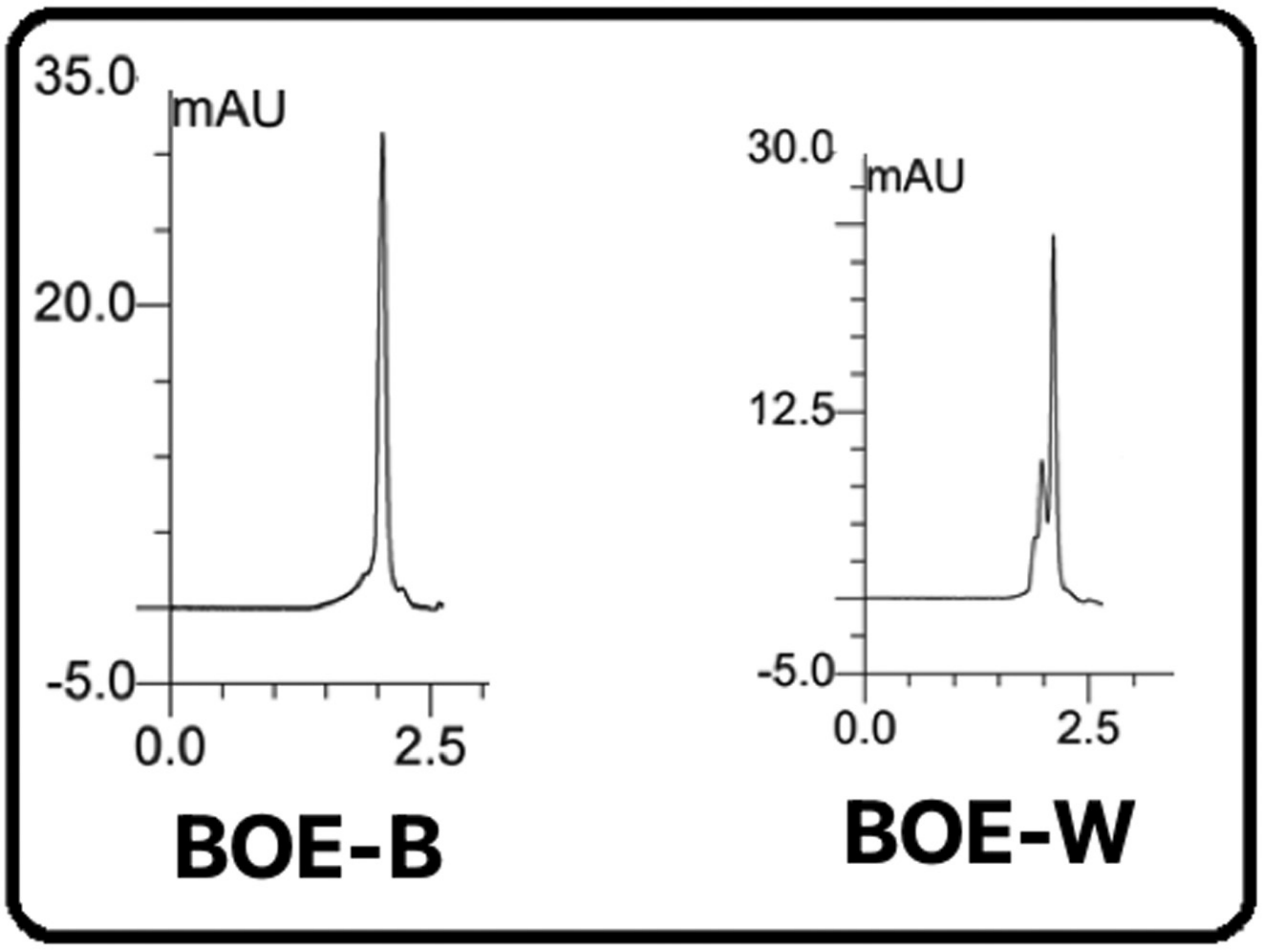
Related to BOE‐W and BOE‐B of Romania HPLC.

Active medicinal substances X and Y can be present in all three models. So, the extract of white cauliflower BOE‐B and doxycycline have similar effective ingredients. Figure 4 shows the HPLC general spectrum for a BOE‐W, BOE‐W, and doxycycline sample. Figure 4a shows the chromatographic profile of BOE‐WB and doxycycline under study. The retention time of significant peaks is presented in Table 4. According to the comparison of entry and exit time in two samples of BOE‐WB and doxycycline, the active ingredients of doxycycline may be present in the two extracts. Figure 5 displays the Romanian BOE‐WB chromatographic view field. (Muntean et al., 2021; Tahmasebi & Esmaeili, 2023). The entry and exit times of these samples were very close to the entry and exit times of the extracts of this research (Figure 4).

### 3.6 Statistical analysis

A two‐way factorial design was used to investigate the effect of extract and drug on growth inhibition halo diameter in different indicators. First, using Lone's test, the equality of the variance of the scores among the subgroups has been checked. If the assumption of the equality of the variance of the scores is confirmed (sig < .05), the factorial design test is reported based on the equality of this assumption. Otherwise, robust tests and corrections will be used (Esmaeili & Hadad, 2015).

Table 5 shows the results of Lon's test for equality of variance of scores for different indicators in different subgroups. It can be seen that the significance level of Lune's test for the scores of *E. coli, P. aeruginosat, S. mutans, S. enterica, and B. cereus* is more than 0.05, so for these variables, the null hypothesis of the confirmation test and the scores have homogeneous variance. The two‐way factorial design is reported based on accepting this assumption (Esmaeili & Gholami, 2015). Still, for the *S. aureus* score, the significance level is <.05, so the factorial design for this variable, the correction effect, and robust tests are used. The results of the two‐way factorial design for the impact of the extract and the drug on the diameter of the halo of growth inhibition in different indicators are presented in Table 6.

**TABLE 5:** The results of Lune's test for the homogeneity of the variance of scores in subgroups.

Variable	Test statistics	Degree of freedom 1	Degree of freedom 2	Meaning surface (sig)
*E. coli*	0.712	11	24	.716
*S. aureus*	3.629	11	24	.004
*P. aeruginosa*	2.012	11	24	.074
*S. mutans*	1.389	11	24	.241
*S. enterica*	0.219	11	24	.994
*B. cereus*	0.830	11	24	.614

**TABLE 6:** Summary of the two‐way factorial design results for the effect of the extract and the drug on the growth inhibition halo diameter in different indicators.

Indicator	Source of changes	Sum of squares	Degrees of freedom	Average of squares	Test statistic (*F*)	Significant level	Effect size
*E. coli*	Fixed effect	8742.250	1	8742.250	15,352.244	.000	0.998
	Extract effect	2881.806	3	960.602	1686.911	.000	0.995
	Medicine effect	222.167	2	111.083	195.073	.000	0.942
	Reaction	98.611	6	16.435	28.862	.000	0.878
	Error effect	13.667	24	0.569	–	–	–
	Total effect	11,958.500	36	–	–	–	–
*S. aureus*	Fixed effect	5220.062	1	5220.062	11,564.446	.000	0.999
	Extract effect	6705.187	3	2235.062	1580.173	.000	0.998
	Medicine effect	13.542	2	6.771	15.000	.000	0.556
	Reaction	40.625	6	6.771	15.000	.000	0.789
	Error effect	10.833	24	0.451	–	–	–
	Total effect	11,990.250	36	–	–	–	–
*P. aeruginosa*	Fixed effect	10,937.674	1	10,937.674	16,071.684	.000	0.999
	Extract effect	3226.187	3	1075.396	1580.173	.000	0.995
	Medicine effect	576.514	2	288.257	423.561	.000	0.972
	Reaction	262.542	6	43.757	64.296	.000	0.944
	Error effect	16.333	24	0.681	–	–	–
	Total effect	15,019.250	36	–	–	–	–
*S. mutans*	Fixed effect	6320.250	1	6320.250	6791.910	.000	0.996
	Extract effect	1350.139	3	450.046	483.632	.000	0.984
	Medicine effect	178.167	2	89.083	95.731	.000	0.889
	Reaction	120.611	6	20.102	21.602	.000	0.844
	Error effect	22.333	24	0.931	–	–	–
	Total effect	7991.500	36	–	–	–	–
*S. enterica*	Fixed effect	8487.016	1	8487.016	11,723.072	.000	0.998
	Extract effect	1314.352	3	438.117	605.169	.000	0.987
	Medicine effect	36.948	2	18.474	25.518	.000	0.680
	Reaction	19.122	6	3.187	4.402	.004	0.524
	Error effect	17.375	24	0.724	–	–	–
	Total effect	9874.813	36	–	–	–	–
*B. cereus*	Fixed effect	8579.391	1	8579.391	9341.643	.000	0.997
	Extract effect	1101.977	3	367.326	399.962	.000	0.980
	Medicine effect	38.198	2	19.099	20.796	.000	0.634
	Reaction	28.705	6	4.784	5.209	.001	0.566
	Error effect	22.042	24	0.918	–	–	–
	Total effect	9770.313	36	–	–	–	–

According to Table 6 and the result of the two‐way factorial design for the *E. coli* variable, it can be seen that the effects of extract (*f* = 1686.911 sig < .05) and medicine (*f* = 195.73 sig < .05) on growth inhibition halo diameter are significant. This means there is a substantial difference between the types of extracts and the types of drugs on the diameter of the halo of growth inhibition in the *E. coli* index. According to the graphs, descriptive statistics, and Tukey's post hoc test, the highest average diameter of the halo of growth inhibition was for doxycycline, the second rank is related to ampicillin, the third rank was associated with BOE‐B, and the last rank was BOE‐W. In the case of drugs, the highest average diameter of the halo of growth inhibition for the drug was 1000 units, the second rank was 500, and the third rank was 250. The same statistical work was done for another case of the plant (Dadashi & Esmaeili, 2021).

According to Table 6 and the result of the two‐way factorial design for the *S. aureus* variable, it can be seen that the effects of extract (*f* = 4951.523 sig < .05) and drug (*f* = 15.000 sig < .05) on growth inhibition halo diameter are significant. This means there is a substantial difference between the types of extracts and the types of drugs on the diameter of the halo of growth inhibition in the *S. aureus* index. According to the graphs, descriptive statistics, and Tukey's post hoc test, the highest average diameter of the halo of growth inhibition was for doxycycline. The second rank is related to ampicillin and ranked third on the same level as BOE‐B and BOE‐W. In the case of medicine, the highest average diameter of the halo of growth inhibition for the drug was 1000 units. The second rank was at a dose level of 500 and a dose of 250.

According to Table 6 and the result of the two‐way factorial design for *P. aeruginosa* variable, it can be seen that the effects of extract (*f* = 1580.173 sig < .05) and drug (*f* = 423.561 sig < .05) are significant on growth inhibition halo diameter. This means there is a substantial difference between the types of extracts and the types of drugs on the growth inhibition halo diameter in the *P. aeruginosa* index. According to the graph's descriptive statistics and Tukey's post hoc test, the highest average diameter of the growth inhibition halo was for doxycycline. The second rank is related to ampicillin and ranked third on the same level as BOE‐B and BOE‐W. In the case of drugs, the highest average diameter of the halo of growth inhibition for the drug was 1000 units, the second rank was 500, and the third rank was 250.

According to Table 6 and the result of the two‐way factorial design for the *S. mutans* variable, it can be seen that the effects of the extract (*f* = 483.632 sig < .05) and the drug (*f* = 95.731 sig < .05) have a significant impact on growth inhibition halo diameter. This means there is a substantial difference between the types of extracts and the types of drugs on the growth inhibition halo diameter in the *S. mutans* index. According to the graphs and, descriptive statistics and Tukey's post hoc test, the highest average diameter of the growth inhibition halo was for doxycycline, the second rank was related to ampicillin, the third rank was associated with BOE‐B and BOE‐W. In the case of medicine, the highest average diameter of the halo of growth inhibition for the drug was 1000 units, the second rank was 500, and the third rank was 250.

According to Table 6 and the result of the two‐way factorial design for the *S. enterica* variable, it can be seen that the effects of extract (*f* = 605.169 sig < .05) and drug (*f* = 25.518 sig < .05) on growth inhibition halo diameter is significant. This means there is a substantial difference between the types of extracts and the types of medicine on the growth inhibition halo diameter in the *S. enterica* index. According to the graph's descriptive statistics and Tukey's post hoc test, the highest average diameter of the growth inhibition halo was for doxycycline. The second rank is related to ampicillin and ranked third on the same level as BOE‐B and BOE‐W. In the case of drugs, the highest average diameter of the halo of growth inhibition for the drug was 1000 units, the second rank was 500, and the third rank was 250. The same scale was used for the anti‐epileptic drug. Statistically, the accuracy of this work was higher than the previous one (Esmaeili & Singh, 2017).

According to Table 6 and the result of the two‐way factorial design for the *B. cereus* variable, it can be seen that the effects of the extract (*f* = 399.692 sig < .05) and the drug (*f* = 20.796 sig < .05) on the growth inhibition halo diameter are significant. This means there is a substantial difference between the types of extracts and the types of drugs on the growth inhibition halo diameter in the B. cereus index. According to the graphs and, descriptive statistics and Tukey's post hoc test, the highest average diameter of the growth inhibition halo was for doxycycline, the second rank is related to ampicillin, the third rank was associated with BOE‐B and BOE‐W. In the case of medicine, the highest average diameter of the halo of growth inhibition for the drug was 1000 units; the second rank was at a dose level of 500 and a dose of 250.

## 4 CONCLUSION

BOE‐WB is low in fat and carbohydrates and high in fiber and vitamin B9, water, and vitamin C. BOE contains several phytochemicals that benefit human health. Sulforaphane is a compound that protects the body against cancer.

Following the resistance of pathogenic bacteria to antibiotics, researchers are searching for new herbal medicine. The results of a GC–MS test on BOE‐WB and identifying oxygenated compounds show that BOE‐B has a higher percentage of oxygenated compounds. For example, 9,12,15‐Octadecatrienal constitutes 72.10% of all BOE‐B compounds. The compounds show their high antioxidant properties from the research done on Romanian and Iraqi BOE. The results were very close to me. It shows the climatic conditions of each country. The antimicrobial test results performed on BOE‐WB showed that it is very close to Gram‐negative and even better than Gram‐positive bacteria. The results of the doxycycline test showed that Gram‐negative bacteria have less resistance (30.33 mm) than Gram‐positive bacteria (27 mm). Finally, the lethal effect of broccoli extract on the strains is very close to doxycycline and can be introduced as a new antimicrobial drug to the medical world. By performing the HPLC test on the medicine and two extracts, the entry and exit times of the samples were very similar. There is a possibility of them being effective medicinal substances.

## AUTHOR CONTRIBUTIONS

**Mahboubeh Taherkhani:** Conceptualization (equal); software (equal).

## ACKNOWLEDGEMENTS

The article's authors thank Dr. Ali Jozi, Director of the Pharmaceutical Chemistry Department, for his efforts.

## FUNDING INFORMATION

This research did not have any funded.

## CONFLICT OF INTEREST STATEMENT

The authors declare no conflict of interest.

## Supporting information


File S1

## Data Availability

The data supporting this study's findings are available on request from the corresponding author.
