# Supplementary material for: Antimicrobial Potential of Brassica oleracea Extracts (White and Broccoli) and Their Resistance Compared to Doxycycline Against Gram‐Positive and Gram‐Negative Bacteria
Source: Food Sci Nutr. 2024 Jul 28;12(10):7492–503. doi: 10.1002/fsn3.4345 (PMC11521745; doi:10.1002/fsn3.4345)
Supplement: Supplementary file 1 — File S1 [file FSN3-12-7492-s001.docx]

**Table S1.** Percentage composition of the BOE-W.

| **KI** | **%** | **Compound** | **No.** |
| --- | --- | --- | --- |
| 952 | 2.80 | Methanethiosulfinate | 1 |
| 955 | 4.91 | Trisulfide, dimethyl | 2 |
| 1135 | 1.08 | 1-Methyl-2-cyano-2-(2'-propyl)-3-ethyl-3-piperideine | 3 |
| 1174 | 1.00 | Cyclotrisiloxane, hexamethyl- | 4 |
| 1216 | 3.74 | Phenyl pyrroline | 5 |
| 1320 | 1.56 | Cyclohexene | 6 |
| 1354 | 2.55 | Dihydrocoumarin | 7 |
| 1378 | 2.92 | Methylbenzene | 8 |
| 1400 | 1.00 | Tetramethylbenzo)indole | 9 |
| 1429 | 12.61 | (2-Methyl-but-3-enyl-2-oxy)-dimethyl-silane | 10 |
| 1435 | 3.78 | Naphthalene | 11 |
| 1439 | 7.16 | (2-Methyl-but-3-enyl-2-oxy)-dimethyl-silane | 12 |
| 1484 | 2.42 | Silane, trimethyl | 13 |
| 1503 | 0.98 | Mannitol | 14 |
| 1527 | 0.98 | Hexasiloxane | 15 |
| 1590 | 3.75 | Tetrahydrobenz[c]azepine | 16 |
| 1599 | 1.55 | Trimethyl-bicyclo[4.1.0]hept-2-en-3-yl)-butan-2-one | 17 |
| 1608 | 3.84 | (5-Nitrohex-1-enyl) benzene | 18 |
| 1624 | 3.25 | Carbazole | 19 |
| 1761 | 1.36 | Dithioisoindoline | 20 |
| 1784 | 1.10 | Pyrimidine | 21 |
| 1804 | 1.24 | Thienyl pyrimidine | 22 |
| 1909 | 1.09 | Glucitol | 23 |
| 1941 | 6.98 | Palmitic acid | 24 |
| 1977 | 1.85 | Ethyl palmitate | 25 |
| 2107 | 1.33 | F]quinoxaline | 26 |
| 2139 | 3.46 | Linolenic acid | 27 |
| 2268 | 2.03 | Ethylhexyl ester | 28 |
| 2303 | 1.82 | Diphenyl phosphate | 29 |
| 2333 | 6.14 | Ethylhexyl isophthalate | 30 |
| 2373 | 5.01 | Nonacosane | 31 |
| 2404 | 2.87 | Phenyl telluride | 32 |
| 2534 | 1.84 | Ergost | 33 |

**Table S2.** Percentage composition of the BOE-B compounds.

| KI | (%) | Compound |
| --- | --- | --- |
| 915 | 0.69 | Furanone, 5-methyl- |
| 951 | 0.63 | methanethiosulphinate |
| 963 | 1.83 | Trisulfide, dimethyl |
| 973 | 0.67 | sec-butyl isopropyl |
| 1148 | 1.69 | Oxime-, methoxy-phenyl- |
| 1197 | 0.82 | Dihydro-benzofuran |
| 1216 | 0.57 | Trimethyl silyloxy ethane |
| 1287 | 1.47 | Methoxy vinyl phenol |
| 1320 | 0.48 | bis(trimethylsilyl)peroxide |
| 1393 | 1.71 | Ribopyranoside, methyl |
| 1429 | 1.39 | Dimethyl-silane |
| 1493 | 0.28 | Cyclo trisiloxane |
| 1526 | 1.51 | -pyrazolium Hydroxide |
| 1590 | 3.33 | Nonanoic acid |
| 1723 | 5.41 | Benzyl benzoate |
| 1847 | 0.30 | Pentyl acetoacetate |
| 1860 | 0.49 | Xycaine |
| 1942 | 5.48 | Hexadecanoic acid |
| 1977 | 3.87 | Ethyl palmitate |
| 2014 | 0.47 | Thiosulfuric acid |
| 2048 | 0.73 | Heptadecanoic acid |
| 2072 | 0.29 | Linolenic acid |
| 2095 | 0.88 | Phytol |
| 2108 | 10.72 | Octadecatrienal |
| 2136 | 3.02 | Ethyl linoleate |
| 2140 | 8.60 | Linolenic acid |
| 2157 | 1.07 | Ethyl Oleate |
| 2180 | 0.37 | Ethyl stearate |
| 2268 | 0.42 | 2-Propenoic acid |
| 2329 | 0.28 | Benzenamine, N-phenyl- |
| 2370 | 0.54 | Eicosatetraenoate |
| 2428 | 0.28 | Eicosane |
| 2478 | 18.95 | Nonacosane |
| 2527 | 0.28 | Dithioisoindoline |
| 2577 | 9.92 | Nonacosanone |
| 2625 | 2.50 | Nonadecene |
| 2675 | 0.86 | Ergost |
| 2725 | 5.10 | Stigmasterol |
| 2785 | 0.30 | Dithioisoindoline |

**
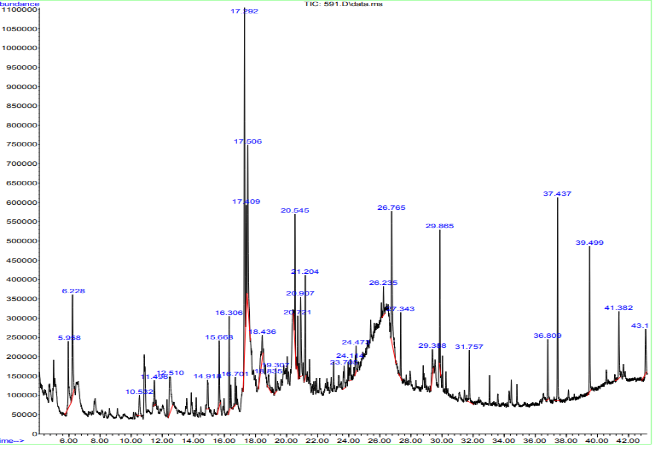
**

**Figure 1S.** GC-MS of (A) BOE-W.


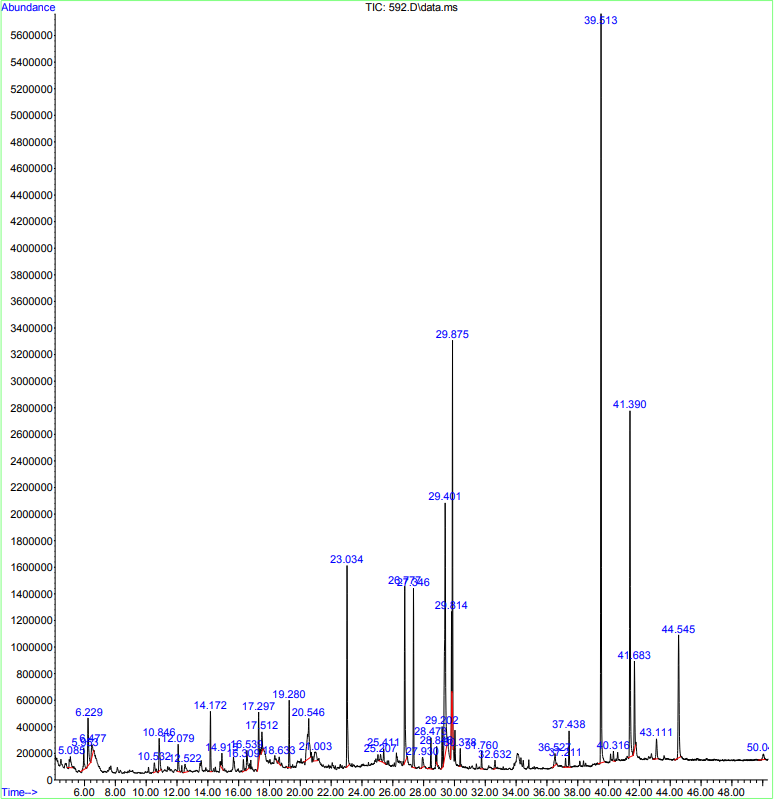


**Figure 2S.** GC-MS of (A) BOE-B.
